# Is the legal framework by itself enough for successful WHO code implementation? A case study from Ethiopia

**DOI:** 10.1111/mcn.13059

**Published:** 2020-08-25

**Authors:** Arnaud Laillou, Heran Gerba, Meseret Zelalem, Dereje Moges, Wendafrash Abera, Tesfaye Chuko, Betre Getahun, Hilemicael Kahsay, Stanley Chitekwe

**Affiliations:** ^1^ Nutrition Section UNICEF Ethiopia Addis Ababa Ethiopia; ^2^ Ethiopian Food and Drug Administration Addis Ababa Ethiopia; ^3^ Department for Maternal, Child Health and Nutrition Federal Ministry of Health Addis Ababa Ethiopia; ^4^ Independent Legal Consultant Addis Ababa Ethiopia

**Keywords:** code, Ethiopia, legislation, violation

## Abstract

Since 2016, Ethiopia has passed several proclamations and directives to regulate the promotion of commercial breastmilk substitute (BMS). Ethiopia's market potential will undoubtedly be the gravitating point for international infant formula companies due to growing urbanization, purchasing power, population, and the relatively low use of BMS to‐date. The aim of this review is to assess the strengths and weaknesses of the existing laws, standards, and monitoring documents used to regulate the marketing of BMSs in Ethiopia and make future recommendations. The study findings highlighted that the regulation on marketing are comprehensive and strong to limit the promotion of infant formula. On the other hand, the regulation on marketing of follow‐up formulas, complementary foods, and growing‐up milk by manufacturers and distributors, media houses, and communication and advertisement agencies are underregulated, especially with regards to the international 69.9 regulation. The monitoring and enforcement of the existing marketing regulations remain limited in the absence of a formal coordination mechanism. Several violations of the national BMS regulations were observed. Forty‐one percent of mothers reported observing the BMS advertising and logos were detected in 36% of health facilities assessed. In 100% of cases, the infant formula labels contained violations. As the lead national authority mandated to regulate food safety, the Ethiopian Food and Drug Authority needs to update its regulations related to the marketing of BMS to fill the loopholes and revise the national law in line with the international code of marketing of BMSs to protect breastfeeding.

**Key Messages**

- Despite existence of legislation, companies are pervasively promoting breast‐milk substitutes in Addis Ababa, particularly on television, as 41% of the interviewed mothers have seen breastmilk substitute advertising.
- The national laws largely exclude the International Code and Monitoring of Breastmilk Substitute (the Code)–related marketing restrictions required to regulate the promotion and advertisement of follow‐up formulas, complementary foods, and growing‐up milks.
- A specific monitoring tool kit of approved legislation needs improvement and implementation.
- Future amendments to regulations should emphasize advertising in order to protect breastfeeding for the nutrition and health of infants and young children as the Ethiopian market becomes more favourable to international trade, including companies that manufacture infant formula.

## 1 INTRODUCTION

Globally, 595,379 childhood deaths per year are attributed to lack of breastfeeding with annual global economic losses estimated between US$257 billion and US$341 billion (Walters, Phan, & Mathisen, 2019). The scale up of optimal breastfeeding practices could have the single largest impact on child mortality of any preventive intervention (Bhutta et al., 2013). Breastfeeding is a protective factor against several infectious diseases and has a positive impact on neurodevelopment, improving IQ, and reducing the risk of attention deficit disorder and generalized developmental and behavioural disorders (Brahm & Valdés, 2017).

Despite sustaining double‐digit economic growth rates during the last 12 years in Ethiopia, malnutrition remains high, with 37.1% of children under the age of 5 years stunted (Ethiopian Public Health Institute & ICF, 2019). Ethiopia has some of the highest absolute numbers of children who are stunted and/or wasted worldwide (UNICEF, World Health Organization (WHO), & International Bank for reconstruction and Development/The World Bank, 2019). The Cost of Hunger study estimated that the annual costs associated with child malnutrition alone are equivalent to 16.5% of Ethiopia's gross domestic product. Evidence exists to suggest that the effort by Ethiopia to reach middle‐income status is hampered by 67% of the adult population who had suffered from stunting as children (WFP, ECA, & African Union, 2013). Breastfeeding is nearly universal in Ethiopia, and at least 85% of children aged 0 to 2 years have been breastfed with a median duration of predominant breastfeeding of 7.3 months (Ethiopian Public Health Institute & ICF, 2019). Despite the relatively low use of breastmilk substitutes (BMSs) in Ethiopia at the moment, growing urbanization (World Bank Group, 2015), the reduction of the population living below the poverty line from 34% in 2011 to 24% in 2016, and the increase in gross national income from 390USD in 2011 to 790USD in 2016 (World Bank, 2019) make the country a suitable market and potential gravitating point for international BMS companies in Africa. Studies show that the prevalence and duration of breastfeeding is declining and being replaced by formula milk in Ethiopia (Abebe et al., 2019; Dessalegn, Tefera, Eskindir, & Shikur, 2012). Unethical marketing of BMS might influence the Ethiopian social norms in urban areas by making the use of formula more fashionable than breastfeeding and exaggerate the health benefits of formula to appear comparable with breast milk as is observed in many Asian countries (Piwoz & Huffman, 2015). These unethical marketing of BMS reduces breastfeeding and increases the use of BMSs.

In response to the unethical marketing of BMS by infant formula manufacturers, the WHO developed the International Code of Marketing of Breastmilk Substitutes (WHO, 1981) and key resolutions such as the 69.9 regulation (WHO, 2016a), which explicitly include follow‐up formulas and growing‐up milks. This international resolution 69.9 calls on countries to implement the WHO's Guidance on Ending the Inappropriate Promotion of Foods for Infants and Young Children (WHO, 2017). As described by Pereira et al. in 2016, companies that produce follow‐up formulas and growing‐up milks had developed similar packaging and labelling to infant formulas and used those products to promote their brands, creating confusion among consumers (Berry, Jones, & Iverson, 2012; Cattaneo et al., 2015).

Since 2016, the Ethiopian government has adopted several directives such as the “Infant Formula and Follow‐up Formula Directive No. 30/2016” and the “Food Advertisement Directive 33/2016” to support breastfeeding by restricting the promotion of BMS marketed for children less than 2 years of age. However, to date, no breaches to these directives have been reported by Food, Medicine, and Healthcare Administration and Control Authority (FMHACA) and/or other inspectors indicating lack of or poor monitoring. In many countries (Hou et al., 2019), stronger monitoring and enforcement mechanisms have been put in place to ensure that the marketing of BMSs does not undermine breastfeeding.

This study critically reviews the content of legislation, standards, and monitoring documents used to prohibit the unethical marketing of BMSs that undermine breastfeeding in Ethiopia and to identify areas of strength and areas needing improvement to ensure that the prevalence of exclusive breastfeeding can reach 70% by 2020 as stated in the National Nutrition Programme strategy (The Federal Demographic Republic of Ethiopia, 2015). This study sought to assess the exposure by mothers and health facilities to commercial advertising of BMSs in Addis Ababa.

## 2 METHODS

To assess the current legislation and monitoring system, researchers collected data through desk reviews and structured interviews with key in 2019. The key informant interviewee was the main source of qualitative data for the situational analysis of the implementation, monitoring, and enforcement of BMS code in the country. Key informants were selected from the Federal Ministry of Health (FMOH), the Food and Medicine Authority, the Ethiopian Broadcasting Authority, UNICEF, WHO, Save the Children, Alive and Thrive, and relevant regional stakeholders. For the regional and city administration‐level assessments, informants were selected from Regional Health Bureaus, FMHACA, and the Trade and Industry Bureau. The qualitative surveys and desk review examined the available laws, policies, the designated institutions, and available processes for BMS monitoring and enforcement at the national level and at the level of two regional states. The analysis focused on (i) legislative mandates and responsibilities of the core national and regional authorities, (ii) comparison of the international code with Ethiopia's national laws, (iii) relevant laws of national authorities outside of the core institutions, and (iv) legal tools for BMS monitoring and enforcement and their current implementation. All findings were then compared with the key provisions of the Ethiopian legislation according to the International Code of Marketing of Breast‐milk Substitutes within the legal measures described in the WHO report of 2018.

To assess the recall of exposure to advertisement of BMSs by health workers, retailers, and mothers of children less than 24 months of age, the interviewers used questionnaires based on an adaptation of the Net Code Ongoing Monitoring Protocol and Periodic Assessment Protocol (WHO & UNICEF, 2017a; WHO & UNICEF, 2017b). The sample size for this study was calculated to detect a 50% prevalence rate of exposure to advertising within the health system, with a measurement error of ±10%. Using a standard of error of 0.025, a response rate of 10%, and assuming a design effect of 1, a sample size of 107 mothers at discharge was determined. Those mothers with children aged 0–24 months were randomly selected at discharge. Recruitment of participants and data collection were conducted on 110 mothers as no mothers refused to respond to the questionnaires from the 11 largest health facilities (of which 63% were public owned) in Addis Ababa as recommended by the Net Code Ongoing Monitoring Protocol. Sampled facilities were alerted of the data collection approximately 1 week prior to the survey. Survey supervisors worked with the health worker in‐charge to identify those mothers ready for discharge and to randomly select the mothers for interviews. The survey took approximately 2 days per health facility.

In order to assess retail outlets, 11 small stores or pharmacies and 10 large stores (one from each subcity) operating in the capital were selected. As recommended (WHO & UNICEF, 2017a; WHO & UNICEF, 2017b), the 11 small stores or pharmacies were chosen in proximity to each of the 11 selected health facilities. Small stores included corner/convenience stores and neighbourhood stores/kiosks. No pharmacies associated with the health facilities were included. The 10 large stores were selected based on prior knowledge that they would carry most of the relevant products available for sale in Addis Ababa. Large stores included national‐chain grocery stores with high volume of sales. The net code checklists guided the monitoring of (a) labels and packaging of products, (b) point‐of‐sale promotions, and (c) health facility promotional materials and activities. The detailed checklists provided a quick snapshot in Addis Ababa of retail locations, health facilities, and package labels to identify any violation, including inspecting labels and promotional materials.

In order to conduct the assessments in health facilities operating in Addis Ababa, ethical approval was obtained from the Addis Ababa Health Bureau Ethical Review Board. Once the ethical approval was obtained, the interviewers requested the approval to conduct the assessment within the hospitals by the direction and the signed consent from the interviewed mothers.

## 3 RESULTS

### 3.1 Legislative mandates and responsibilities

**FIGURE 1:**
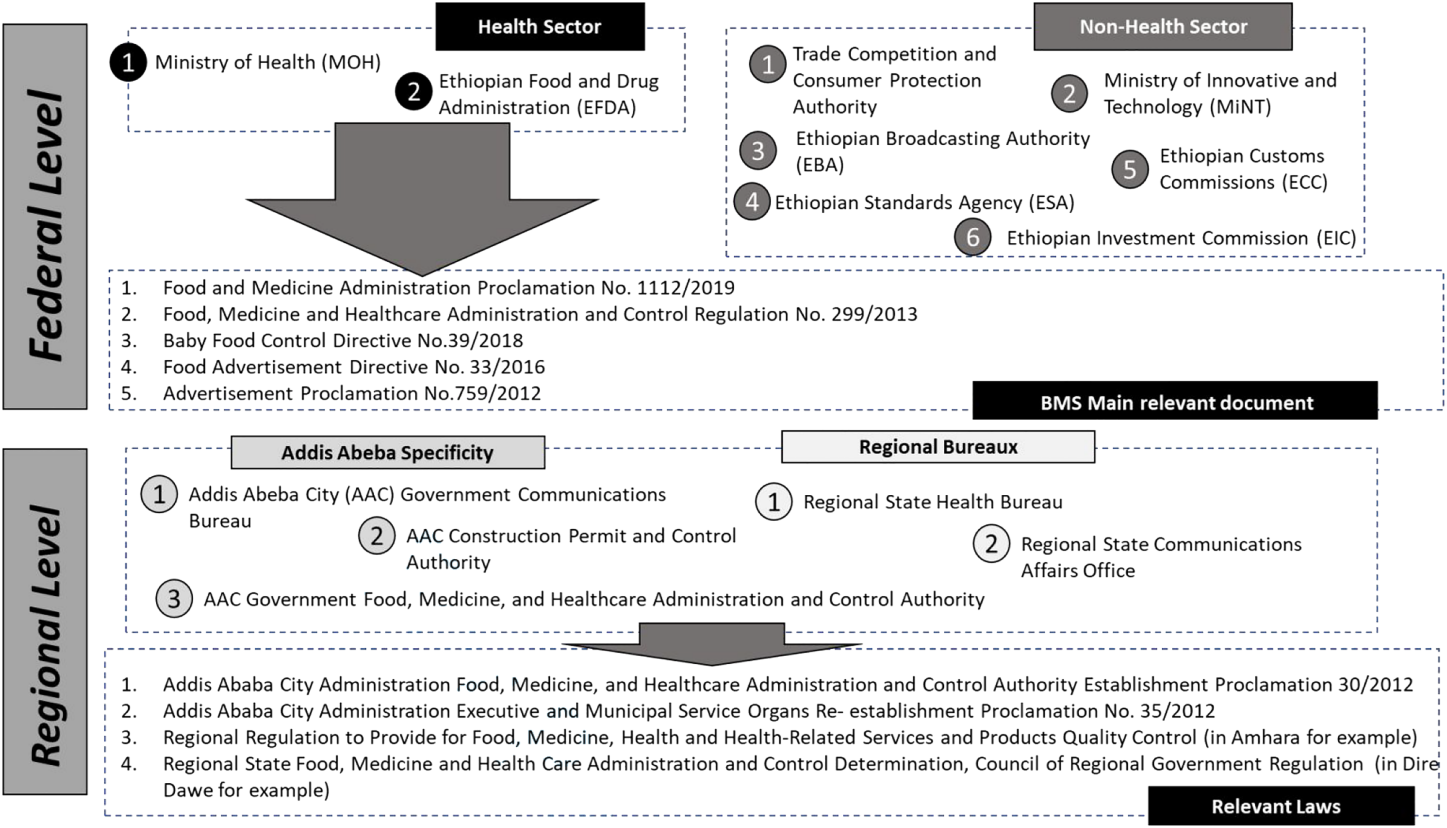
Core national authorities for breastmilk substitute monitoring and related legislation

At the national level, the Ethiopian Food and Drug Administration (EFDA) and the FMOH constitute the core public health authorities (see Figure 1). FMOH has the responsibility to monitor compliance of health institutions owned by the federal government, alternative and complementary medicine practitioners, and other international health professionals practicing in Ethiopia. The EFDA has the responsibility to regulate the conduct of business by manufacturers and distributors, importation, and interstate trade of BMS in Ethiopia. EFDA enforces regulations related to the advertisement of BMS, whereas the Ethiopian Broadcast Authority ensures that the advertisement of BMS through TV and radio channels are following the regulation. Other nonhealth authorities are part of the landscape of organizations that ensure enforcement of directives. For example, the Ethiopian Custom Commissions conducts compliance inspections and releases registered BMS products before their importation into Ethiopia. The Ethiopian Standard Agency issues national compulsory standards on the content and labelling of infant formula, processed cereal‐based baby food, and other BMS. The Ethiopian BMS regulation is articulated towards three legislative texts: (i) the Food Advertisement Directive (33/2016), (ii) the Baby Food Control Directive (39/2016 and amended in 2018), and the Food and Medicine Administration Proclamation (1112/2019).

Due to Ethiopia's federal configuration, the Regional Health Bureau and the Regional State Communications Bureau regional bureaus also have the mandate to implement the mandates around the regulation of BMS such as advertisement, marketing, promotion to health professionals, and registration of health care workers. They regulate most health workers/facilities and any advertisement and promotion that are local in nature and does not transcend regional border.

### 3.2 Gaps and bottlenecks in current regulation and monitoring tools

The Ethiopian government banned the marketing of infant formula through any means of advertisement, passed legislation on the labelling requirements, and provided a definition of the products included in those legal frameworks since 2016. The proclamation 1112/2019 and directives 33/2016 and 39/2018 administrated by EFDA and other agencies have several loopholes which limit the optimal implementation of the national code in Ethiopia to ensure minimal to no violation. For example, bottle feeding and teats are not even covered in the scope of the national laws and regulations; thus, advertising of follow‐up formula, growing‐up milk, complementary feeding through bottles, pacifiers, and teats samples is not banned.

**FIGURE 2:**
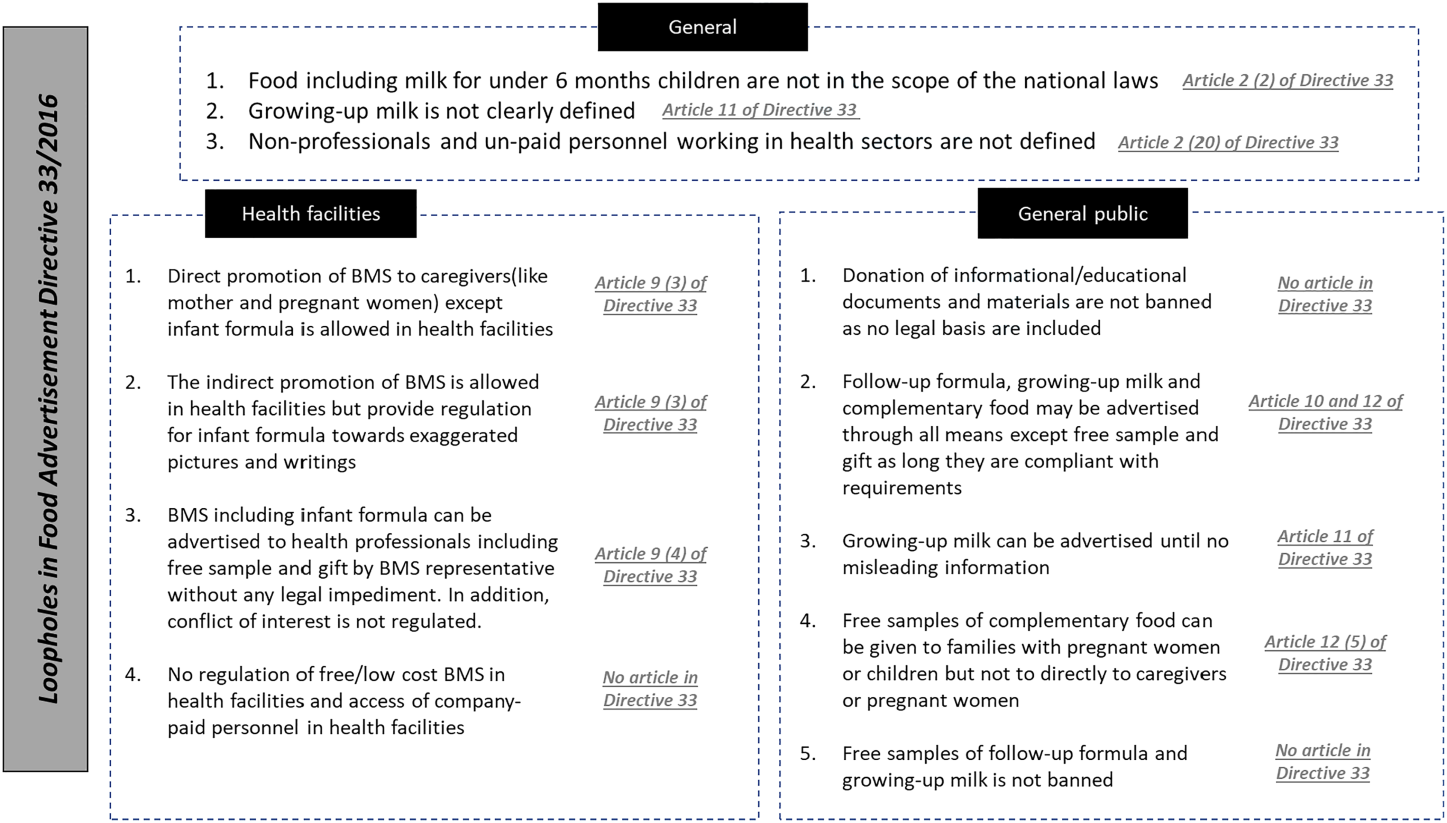
Bottlenecks in the food advertisement Directive 33

The most critical bottlenecks on advertisement under Directive 33 are presented in Figure 2. To date, the highest restrictions are on public promotion and issuing as gifts to caregivers of infant formula compared with other product categories which also could reduce breastfeeding continuation until the child reaches 2 years old. Table 1 presents the key provisions of the Ethiopian legislation according to the international code of marketing of breast‐milk substitutes within legal measures, whereas Table 2 addresses the number of violations observed by type of retail.

**TABLE 1:** Key provisions of the Ethiopian legislations according to the international code of marketing of breast‐milk substitutes within legal measures

Provisions with legal measures	Provisions under Ethiopian Laws
**Products covered**	
Infant formula	Yes
Follow‐up formula	Yes
Complimentary food	Yes
Feeding bottles, teats, and pacifiers	Yes
Milk for mothers	Yes
Other designated products	Yes
**Milk products covered up to age**	Not clear
**Complementary foods covered up to age**	Not clear
**Informational/educational materials covered**	No
**Required information for informational/educational materials**	
Benefit and superiority of breastfeeding	No
Maternal nutrition and preparation for and maintenance of breastfeeding	No
Negative effect on breastfeeding or bottle‐feeding	No
Difficulty reversing the decision not to breastfeed	No
Proper use of infant formula	No
**Required information for materials on breastmilk substitute**	
Social and financial implication	No
Health hazards of inappropriate feeding	No
Health hazards of inappropriate use	No
**Prohibition of pictures/text idealizing breastmilk substitute**	Partial restriction
**Approval required for donation of company materials**	No
**Prohibitions of promotion to the general public**	
Advertising	Partial restriction
Sales device	Some restrictions
**Sample and gift, and contact with mothers**	Yes
**Prohibitions of promotion to health workers/facilities**	
Provision of low‐cost supplies	No
Materials and gifts	
**Required information on labels of breastmilk substitutes**	
Recommended age of introduction	No
Message on the superiority of breastfeeding	Yes
Only to be used on the advice of health workers.	Yes
Preparation instructions	Yes
Bans of pictures/text idealizing infant formula	Yes
Warning on pathogenic microorganisms	No
Ban on nutrition and health claims	Yes
**Criteria for monitoring mechanism**	
Mandates monitoring mechanism	No
Independent and transparent	No
Free from commercial interest	No
Empowered to investigate code violation	No
Empowered to impose a sanction	No

**TABLE 2:** Observation of none‐compliance in Addis Ababa

Article	Observation of none‐compliance	Details
Article 4: Information and education		
Sub‐rticle 4.2. Informational and educational materials, whether written, audio, or visual, dealing with the feeding of infants and intended to reach pregnant women and mothers of infants and young children	36% of health facilities 24% of retailers	All the informational/educational materials found at the health facilities and retail outlets were created by BMS manufacturers or distributors. None of them included the minimum necessary information
Subarticle 4.3. Companies that market foods for infants and young children should not create conflict of interest in health facilities or throughout health systems	27% of health facilities	The equipment observed with logo from BMS manufacturers were infant weight scale, working cloth, and penholder
Article 5: The general public and mothers		
Subarticle 5.1. There should be no advertising or other forms of promotion to the public of products within the scope of the Code	41% of the mothers interviewed	They reported seeing at least one BMS promotion in the past 6 months: 73.8% on TV and 19.7% on billboard. Only 50% of the television incorporated the BMS code provisions into their media monitoring policy and 38% auto reported BMS advertisement into their channel
Subarticle 5.2. Manufacturers and distributors should not provide to pregnant women, mothers, or members of their families, sample of products	0% of the mothers interviewed	‐
Subarticle 5.3. No point‐of‐sale advertising, giving of samples, or any other promotion device to induce sales directly to the consumer at retail level	16 violations	62.5% were poster on display and 19% discount to consumers
Subarticle 5.4. No distribution to pregnant women or mothers of children any gifts of articles or utensils that may promote the use of BMS	0% of the mothers interviewed	‐
Subarticle 5.5. Marketing personnel, in their business capacity, should not seek direct or indirect contact of any kind with pregnant women or with mothers of infants and young children	21% of health workers 29% of mothers	The 7 health workers reported 17 reports of such contact made by baby food companies. The mothers reported that they were advised to feed their baby any milk products other than breast milk in the past 6 months
Article 6: Health care system		
Subarticle 6.2. No facility of health care system should be used for prompting infant formula or other products	6% of mothers	Mothers reported a health worker telling them to use a local commercial baby food/drink product based in Addis Ababa
Article 7: Health workers		
Subarticle 7.2. Information provided by manufacturers and distributors to health professionals regarding products should be restricted to scientific and factual matters	36% of health facilities	All the observed materials were non‐compliant as per any of the subitems under Subarticle 4.2 as well as Subarticle 7.2
Subarticle 7.3. No financial or material inducements to promote products should be offered by manufacturers or distributors to health workers or members of their families or should be accepted	15% of health workers	BMS company representatives made offers to sponsor events or workshops for health facility/staff as well as provided invitation and/or support to attend events/workshops outside the health facility
Subarticle 7.4. Samples of BMS, or of equipment or utensils for their preparation or use, should not be provided to health workers. Health workers should not give samples of infant formula to pregnant women, mothers of infants and young children, or members of their families	0% of health workers	‐

Abbreviation: BMS, breastmilk substitute.

National and regional authorities within their jurisdiction have comprehensive enforcement power, which includes (i) entering into a licensed institution and inspect regulated products and raw materials at port of entry and exit, (ii) conducting investigations and ordering temporary closures of noncompliant businesses institutions, (iii) detention and seizure of any product, and (iv) ordering of laboratory examination. A panel called a “health regulatory panel” within the EFDA was established under the directive to review fairness and legality of the various enforcement actions and has the power to overturn any decision made by the technical unit of EFDA. The national and regional public health authorities (EFDA and MOH health inspectors) use generic checklists and quality audit tools for the monitoring and enforcement of BMS‐related laws. These generic checklists do not have specific indicators on marketing, advertisement, and promotion activities which limits the scope of the enforcement. During the interviews, there was no evidence of inspectors submitting monthly reports summarizing their monitoring activities to their respective ministry or departments at both national and subnational levels. The collaboration between the EFDA, EBA, and others on the regulation of national code of marketing of BMS remains weak. The EFDA, which is the leading national authority on the Code of marketing of BMS, argues that the weak coordination among federal government authorities is due to the absence of a formal monitoring mechanism. Even though platforms such as the Joint Steering Committee exists at national and regional levels, to prepare, monitor, and evaluate the implementation of the national health plan and other activities, it cannot substitute national code‐specific coordinating bodies.

### 3.3 Assessment of baby food companies' compliance

**FIGURE 3:**
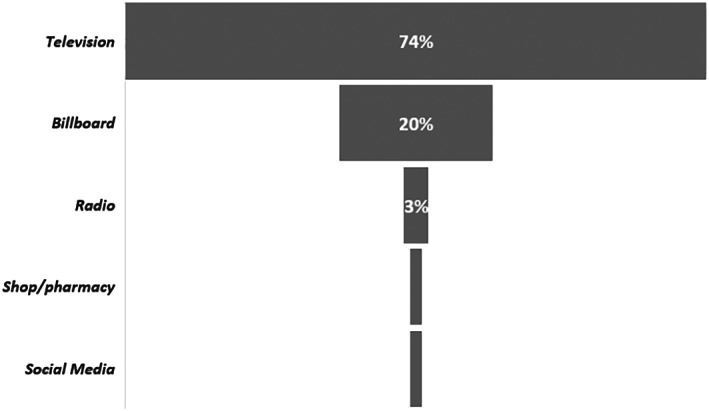
Type of breastmilk substitute promotion according to 110 mothers assessed

**FIGURE 4:**
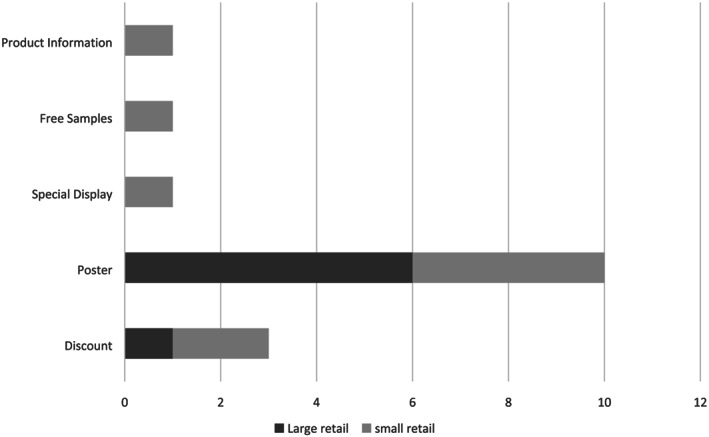
Numbers of irregularities observed in 21 assessed retails from Addis Ababa

Table 2 shows the violations of the international code of BMS in Addis Ababa among 11 health facilities, 33 health workers, 110 mothers, eight national TV broadcast agencies, and 21 retail outlets specified by of the reference articles. Most of the reported violations were made through the television and posters at retail level as presented in Figures 3 and 4.

A review of product labels was conducted for 30 unique products produced by different baby food companies. Each label assessed had at least one labelling violation. One hundred fifteen infractions were observed on those 30 labels according to Subarticles 9.2 and 9.4 of the national code, as well as WHA 58.32. For example, 42% of the infant formulas' samples were missing a statement that the product could only be used under the recommendation of health worker, and 23% did not include a statement of the superiority of breastfeeding on their product label.

## 4 DISCUSSION

This paper shows the structure and how the Ethiopian government expressed its political aspiration to protect and promote optimal breastfeeding by adopting several generic proclamations and two national implementing directives on the Code of Marketing of BMSs supported by regional mechanisms. As shown by Michaud‐Létourneau, Gayard, and Pelletier (2019), the government of Ethiopia has really taken on the responsibility of improving the national code and is collaborating with development partners to achieve a common goal. Unfortunately, the national laws largely overlook the International Code and Monitoring of Breastmilk Substitute (the Code)–related marketing restrictions on the promotion and advertisement of follow‐up formula, complementary foods, and growing‐up milks by manufacturers and distributors, media houses, and communication and advertisement agencies including the international recommendation 69.9 (WHO, 2016b). Even though the consumption of such products seems low (7.6% for children aged 0–5 months and 3.1% for children aged 6–9 months) according to the 2019 Ethiopian Demographic Health Survey, this study indicates that promotion of BMSs in Addis Ababa, Ethiopia, appears to be significant as 41% of the interviewed mothers reported seeing at least one BMS advertisement in the past 6 months on television and branding within stores. The significant increase of the use of bottles with a nipple among children 12–23 months of age over the last 3 years from 13.2% (Ethiopian Public Health Institute & ICF, 2016) to 25.3% (Ethiopian Public Health Institute & ICF, 2019) is an early warning of potential threat from BMS or inappropriate use of complementary feeding. As shown in our study, those practices are not constrained by any regulations. National laws do not define bottles, pacifiers, and teats as a designated product for code regulation. Unfortunately, diluted complementary food could easily become a BMS once it is given using a bottle and a nipple. The complexity of defining when a complementary food becomes a BMS was already raised in Indonesia (Soekarjo & Zehner, 2011). Even the indirect promotion of follow‐up formula and growing‐up milk through bottles, pacifiers, and teats is not banned in Ethiopia and should therefore be remedied with future amendments to the existing directives. All these required amendments are presented in Table 3.

**TABLE 3:** Amendments required to complied with the International Code of Breast‐Milk Substitutes and related regulation

Provisions with legal measures	Ethiopian laws	Description
Milk products covered up to age	Not clear	The national law defined growing up milk as any food marketed and suitable for feeding young children from the age of 2 years up to 3 years of age. It appears to deregulate milk products for children between the period of 12 to 24 months. National laws that are applicable on advertisement and promotion matters does not define milk products or growing‐up milk products
Complementary foods covered up to age	Not clear	Contrary to the Code and WHO subsequent resolution, the Food Advertisement Directive mistook the definition of dietary supplement for complimentary food. It defined complementary food as “any foodstuff whose purpose is to supplement the normal diet and which are concentrated sources of nutrients or other substances with a nutritional or physiological effect, and marketed in dose form, like capsules, tablets, pills and other similar forms of liquids and powders designed to be taken in measured quantities” (Directive 33, Article 2(4))
Informational/educational materials covered	No	No national law provides code‐compliant measures on these matters
Required information for informational/educational materials		
Benefit and superiority of breastfeeding	No	No national law provides code‐compliant measures on these matters
Maternal nutrition and preparation for and maintenance of breastfeeding	No	No national law provides code‐compliant measures on these matters
Negative effect on breastfeeding or bottle‐feeding	No	No national law provides code‐compliant measures on these matters
Difficulty reversing the decision not to breastfeed	No	No national law provides code‐compliant measures on these matters
Proper use of infant formula	No	No national law provides code‐compliant measures on these matters
Required information for materials on breastmilk substitute		
Social and financial implication	No	No national law provides code‐compliant measures on these matters
Health hazards of inappropriate feeding	No	No national law provides code‐compliant measures on these matters
Health hazards of inappropriate use	No	No national law provides code‐compliant measures on these matters
Prohibition of pictures/text idealizing breastmilk substitute	Partial restriction	Pictures of the infants or other pictures or texts, which may idealize the use of breastmilk products on its container and the label is banned. (See, Baby Food Control Directive 39/2016, Art 8(e).) Promotion of infant formula only through exaggerated pictures and writings about its use and relevance in the health care institution, is banned. (see Art 9(3), Food Advertisement Directive 36). However, national laws do not ban the idealize the use of breastmilk products, including through educational and informational materials.
Approval required for donation of company materials	No	No code related national law provides the legal basis to regulate the manner for the donations of informational or educational equipment or materials by the BMS industry. The Code requires such donation to be done at the request and with the written approval of the appropriate government authority and that no BMS proprietary name be referred but the placement of the donating company's name or logo. (Article 4.3 of ICMBS)
Prohibitions of promotion to the general public		
Advertising	Partial restriction	The advertisement of infant formula through any advertisement dissemination means is banned. (See, Art 59(4), FAMAP 1112/2019). On the other hand, follow‐up formula and complementary food may be advertised through all advertisement means (except free sample and gift for complementary food. (See Art 10 and 12, Food Advertisement Directive 36)
Sales device	Some restrictions	Posting messages like “reduced price” and “clearance sale” at point of sales to advertise and promote infant formula and complementary food (but other product categories) is banned (see Art 9 ((2) and 12(5), Food Advertisement Directive). National laws prohibit no other sales device besides posting related messages. However, sales devices, including special displays, discount coupons, loss‐leaders, and tie‐in sales are not restricted for all product categories except for infant formula
Sample and gift, and contact with mothers	Partial restriction	Advertisement of infant formula and complementary food through giving product free samples and related gifts and articles to pregnant women, mothers of infant children, and their families are banned under the Food Advertisement Directive. (See Article 9 ((2), Article 12(5), Food Advertisement Directive.) However, Free samples and gifts of BMS, including infant formula to health care workers, are not banned. Giving free samples and related gifts and articles of follow‐up formula and growing up milk to anyone is not forbidden. Providing free associated samples and gifts and items of complementary food to the families of pregnant women, mothers of infants and young children are not banned. (See Art 9, 10, and 12, Food Advertisement Directive)
Prohibitions of promotion to health workers/facilities		
Provision of low‐cost supplies	No	No national law provides code‐compliant measures on these matters.
Materials and gifts	No	Infant formula may be directly advertised to health professionals. National law only regulates the manner of direct promotion. The representatives of manufacturers or distributors may not say that BMS could replace or is superior to breastfeeding. Free samples and gifts of BMS, including infant formula to health care workers, are not banned. All the remaining BMS products can be directly promoted to health workers without any legal impediment. (See Art 9 (4), Food Advertisement Directive.) Financial support and incentives that would create conflicts of interest are not banned or regulated under the Food Advertisement Directive and other national laws
Required information on labels of breastmilk substitutes		
Recommended age of introduction	No	No national law provides code‐compliant measures on these matters
Warning on pathogenic microorganisms	No	No national law provides code‐compliant measures on these matters
Criteria for monitoring mechanism		
Mandate monitoring mechanism	No	The national and regional food safety laws give legal authorities to inspectors and other officers of EFDA and regional health regulators to monitor and enforce food safety laws, including for the marketing and promotion of BMS. No mechanism that involves all the stakeholders is established at the national level. The power to investigate non‐compliance and impose sanctions lies with individual inspectors and the EFDA and regional health regulatory authorities
Independent and transparent	No	
Free from commercial interest	No	
Empowered to investigate code violation	No	
Empowered to impose a sanction	No	

Abbreviations: BMS, breastmilk substitute; EFDA, Ethiopian Food and Drug Administration; the Code, International Code and Monitoring of Breastmilk Substitute.

The use of BMSs negatively impacts exclusive breastfeeding and can shorten the duration of breastfeeding (Sundaram et al., 2013), both of which can have wider effects on the nutrition and health of children and mothers. Therefore, the ban of their promotion is very critical under the International Code. Our study has shown several loopholes in the food advertisement directive 33/2016 which would require urgent actions to protect breastfeeding. Even countries with early regulation, like China in 1995, 20 years later continue to show national code violation with 76.1% of women assessed having received free samples in or near hospitals (Liu, Dai, Xie, & Chen, 2014).

There is need to define more explicitly the following terms in order to improve the existing directives: (i) what is growing‐up milk? and (ii) what is the scope of promotion restriction to and from nonprofessional and/or unpaid personnel working in health facilities? More than 10 bottlenecks were raised by our assessment as shown in Figure 2 and could be easily corrected through fine tuning of some articles in the existing directives (Table 3) and following the 2017 WHO guidance (WHO, 2017). Companies will use any backdoor channels to ensure that their products are being promoted and sold to the potential consumers as was observed in Nepal, Cambodia, Senegal, and Tanzania (Zehner, 2016). With a global sales of breastfeeding substitutes of US$40 billion in 2013 (Piwoz & Huffman, 2015), Ethiopia, with a population of more than 100 million, and ranked in 2015 among the top 20 fastest‐growing economies in the world (Deloitte, 2015), seems to be a target market for BMSs, thus the need to strengthen the national legislation.

In addition, the promotion of many BMS within the health care system directed at health professionals are not regulated in Ethiopia because national laws do not provide specific provisions governing code compliance by health professional and health facilities. Quality audit tools and checklists used by MOH, retail inspectors, and the Ethiopian Broadcast Authority do not have BMS specific compliance indicators. Unfortunately, limited efficient examples of national code monitoring have been documented (WHO, UNICEF, & International Baby Food Action Network, 2016)—only 32 countries report having a formal monitoring system in place and less than a quarter of the countries with a monitoring mechanism publish their results. Only six countries report having dedicated budgets or funding for monitoring and enforcement of the code. Thus, Ethiopia needs both the strengthening of national legislation and improvements in monitoring and compliance of manufacturers, distributors, and retailers who conduct point‐of‐sales promotions and within health facilities as recommended in Cambodia (Champeny et al., 2016). Even though our study did not assess the capacity of inspectors from the Ministry of Health at federal and regional level, there is a need for capacity building to strengthen their ability to detect violations and enforce code‐related laws as described in previous publications (Barennes, Slesak, Goyet, Aaron, & Srour, 2016; Hou et al., 2019). During a qualitative discussion implemented in parallel to our study, many of the food safety inspectors and officers at both national and regional level have limited awareness of the gaps in the relevant national laws and the Code.

## 5 CONCLUSION

In parallel to amending the existing directives in line with the international 69.9 recommendations of the Code, Ethiopia should develop an action plan for the nationwide implementation of the monitoring and enforcement of the directives. Through the action plan, regional inspectors should have a clear understanding of their assigned roles and tasks within the monitoring and enforcement system before beginning their inspections. The monitoring system should include checklists integrated into routine activities to encourage the inspectors to complete checklists, especially as they are expected to enforce the directives. If violators remain noncompliant, Ethiopia should consider taking a stricter approach against noncompliant health centres, broadcast channels, retailers, and BMS suppliers.

## CONFLICTS OF INTEREST

The authors declare no conflict of interest. The opinions and statements in this article are those of the authors and may not reflect official policies or opinions of the organizations they belong to.

## CONTRIBUTIONS

AL, HG, MZ, DM, and TC were responsible for the study design, data analysis, and interpretation. AL, HG, and DM were responsible for drafting the manuscript. BG, HK, and SC reviewewd manuscript. All authors approved the final manuscript.
